# Validation and Clinical Application of a Biopsychosocial Model of Pain Intensity and Functional Disability in Patients with a Pediatric Chronic Pain Condition Referred to a Subspecialty Clinic

**DOI:** 10.1155/2013/143292

**Published:** 2013-10-22

**Authors:** Thomas R. Vetter, Gerald McGwin, Cynthia L. Bridgewater, Avi Madan-Swain, Lee I. Ascherman

**Affiliations:** ^1^Department of Anesthesiology, University of Alabama School of Medicine, JT862, 619 19th Street South, Birmingham, AL 35249-6810, USA; ^2^Department of Epidemiology, School of Public Health, University of Alabama at Birmingham, 1922 7th Avenue South, Suite 120, Birmingham, AL 35294-0022, USA; ^3^Department of Nursing, Children's Hospital of Alabama, 1600 7th Avenue South, Birmingham, AL 35233, USA; ^4^Division of Pediatric Hematology-Oncology, Department of Pediatrics, University of Alabama School of Medicine, ACC 512, 1600 7th Avenue South, Birmingham, AL 35233, USA; ^5^Division of Pediatric Psychiatry, Department of Psychiatry, University of Alabama School of Medicine, EFH H339, 1720 University Boulevard, Birmingham, AL 35294, USA

## Abstract

*Background*. Pediatric chronic pain is considered to be a multidimensional construct that includes biological, psychological, and social components. *Methods*. The 99 enrolled study patients (mean age 13.2 years, 71% female, 81% Caucasian) and an accompanying parent completed a series of health-related questionnaires at the time of their initial appointment in a pediatric chronic pain medicine clinic. *Results*. Significant correlations (*r* ≥ 0.30, *P* < 0.05) were observed between pediatric chronic pain intensity and patient anxiety, patient depression, patient pain coping, parent chronic pain intensity, and parent functional disability. Pediatric chronic pain intensity was significantly associated with patient anxiety (*P* = 0.002). Significant correlations (*r* ≥ 0.30, *P* < 0.05) were observed between pediatric functional disability and patient chronic pain intensity, patient anxiety, patient depression, patient pain coping, parent chronic pain intensity, parent functional disability, parent anxiety, parent depression, and parent stress. Pediatric functional disability was significantly associated with patient chronic pain intensity (*P* = 0.025), patient anxiety (*P* = 0.021), patient pain coping (*P* = 0.009), and parent functional disability (*P* = 0.027). *Conclusions*. These findings provide empirical support of a multidimensional Biobehavioral Model of Pediatric Pain. However, the practical clinical application of the present findings and much of the similar previously published data may be tenuous.

## 1. Introduction

Pediatric chronic pain has extensive and often sustained detrimental effects on the health, development, and quality of life of young people, with a concomitant adverse impact on all those invested in their well-being [1–6].

A Delphi poll of professionals with a specific interest in chronic pain in children and adolescents was undertaken to reach a consensus as to the factors associated with pediatric chronic pain and disability [7]. Factors deemed most important in the development of pediatric chronic pain and disability included (a) child's psychological characteristics: the child's tendency to somaticize, depressed personality, and anxious personality; (b) parent's psychological characteristics: parental emotional instability; (c) characteristics of the pain experience: suffering from constant pain and a family history of chronic pain; (d) characteristics of pain management: an excessive use of healthcare services for the child pain complaints, an inappropriate consumption of medicines to relieve the pain, doctor searching for the pain problem without finding anything wrong, and a low compliance with the healthcare professionals' recommendations; (e) psychological factors related to the child's pain experience: catastrophic thinking of the child and parents about the child's pain, child's negative expectations about the course of her or his pain problems, and the presence of positive reinforcements in response to the child's pain behaviors; and (f) a stressful environment [7].

**Figure 1:**
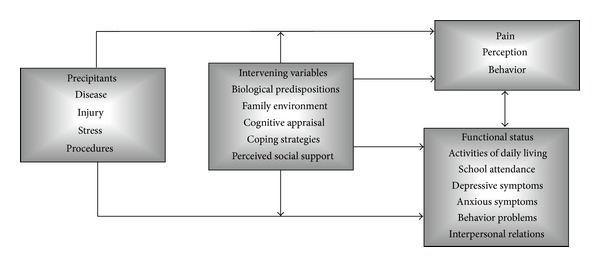
Multidimensional Biobehavioral Model of Pediatric Pain [12].

Current thinking considers chronic pain, including in children and adolescents, to be a multidimensional construct that includes biological, psychological, and social components [8–11]. A multidimensional Biobehavioral Model of Pediatric Pain (Figure 1) [12] was developed by Varni and colleagues in an attempt to account for the observed wide variability in pediatric pain perception, pain behavior, and functional status [13]. This multidimensional biopsychosocial model is predicated on there being a number of potentially modifiable precipitants and intervening factors that contribute to pediatric pain perception and a child's associated functional status and health-related quality of life [14]. This multidimensional Biobehavioral Model of Pediatric Pain has successfully been applied in the management of symptomatic juvenile rheumatoid arthritis [15]. This model has also been applied in the study of headache and abdominal pain in urban early adolescents [16].

This prospective study was undertaken (a) to identify the factors associated with pediatric patients' self-reported chronic pain intensity and functional disability and (b) to further validate the Biobehavioral Model of Pediatric Pain in patients with a variety of chronic pain disorders, who were referred to a dedicated pediatric chronic pain medicine program. Observations are made on the clinical application and ramifications of this and other similar pediatric chronic pain study findings.

## 2. Materials and Methods

### 2.1. Setting and Participants

Study participation was offered to 145 eligible patients, ranging between 8 years and 17 years of age, who were initially and consecutively evaluated in an outpatient pediatric chronic pain medicine clinic, located at a free-standing children's hospital, between May 2009 and December 2010. All patients were directly referred to this ambulatory clinic by their primary care physician or another subspecialist physician, with an existing chronic pain diagnosis. This study was approved by the Institutional Review Board of the University of Alabama at Birmingham and abided by the Ethical Principles for Medical Research Involving Human Subjects outlined in the Declaration of Helsinki. Written parental consent and written patient assent were obtained prior to study enrollment.

Because of the low prevalence in the study population of persons of Hispanic or Latino origin (3.2%) and of a language other than English (e.g., Spanish) being spoken at home (3.2%) [17], patients in whose nuclear families English was not the primary or native language were excluded from this study. This exclusion criterion also was based on the lack of a validated Spanish language version of one of the planned measurement instruments (Functional Disability Inventory). Patients suffering from severe cognitive dysfunction (i.e., mental retardation) and thus unable to complete the patient questionnaires were also excluded.

### 2.2. Study Design

Enrolled patients and their parents completed the study questionnaires, diagnostic instruments, and health surveys (Table 1) at the time of their initial appointment in the pediatric chronic pain medicine clinic, but prior to being evaluated and treated by a pain medicine physician or any other clinic healthcare providers. The patient and the parent were consistently instructed by the study coordinator (C.L.B.) on how to complete the various measurement instruments. The patient and the parent were also explicitly instructed to complete the study questionnaires completely independent, so as to minimize any respondent cross-contamination. All of the study instruments were administered in the same order. Patients and their parents were provided ample time and privacy to complete the study forms.

### 2.3. Sociodemographics and Clinical History

In addition to the patient's and the parent's responses to the series of measurement instruments listed below, data were collected regarding patient age (in years); patient sex (male/female); patient race (African-American/Caucasian/Hispanic/Other); primary chronic pain-related diagnosis (ICD-9 coding); and duration of pain symptoms (in months).

### 2.4. Child/Adolescent Patient Measurement Instruments 

#### 2.4.1. Pain Intensity: Pediatric Pain Questionnaire (PPQ)

The Pediatric Pain Questionnaire (PPQ) is a patient self-reported assessment instrument for children and adolescents (8–18 years old) [18–20]. The PPQ assesses pain intensity with a 100 millimeter horizontal line (Visual Analogue Scale, VAS) that is without numbers but ranges from 0 (anchored either by a smiling cartoon face and “no hurt at all” or by “no pain, not hurting, and no discomfort”) to 100 (anchored either by a sad cartoon face and “hurting a whole lot” or by “severe pain, hurting a whole lot, and very uncomfortable”). The PPQ is a reliable and valid tool for measuring pediatric chronic pain intensity [18, 20]. An evidence-based review of the various assessment tools for pediatric pain, including pain intensity self-report scales, rated the VAS and PPQ as “well-established” [21]. No other sensory or affective elements of the PPQ were utilized for this study.

#### 2.4.2. Disability and Functional Impairment: Functional Disability Inventory

The Functional Disability Inventory (FDI) Child and Adolescent Form is a patient self-reported questionnaire for children and adolescents (8–17 years old), which assesses perceived difficulty in performing activities in the context of school, home, recreation, and social interaction [22, 23]. The FDI consists of 15 items that are each rated on a four-point scale (0 = no trouble, 1 = a little trouble, 2 = some trouble, 3 = a lot of trouble, and 4 = impossible), generating a total score of 0 to 60. The FDI has demonstrated sufficient reliability and validity in assessing disability across the range of chronic pain conditions [24]. Based upon published psychometric properties, a recent evidence-based review classified the Functional Disability Inventory as “well-established” [25]. Of note, a recent multicenter study validated a three-level, ordinal classification system for the FDI (0–12 = “no/minimal,” 13–29 = “moderate,” and ≥ 30 = “severe”), with patients scoring in the “moderate” disability category being the most typical patients presenting to specialty pediatric pain medicine clinics [26]. However, the raw total (0–60) FDI score was used for the present analyses.

#### 2.4.3. Depression: Children's Depression Inventory Form

The Children's Depression Inventory Form (CDI) assesses symptoms of depression in children and adolescents [27, 28]. The presently applied full version of the CDI consists of 27 items, with responses rated on a three-point scale (0 to 2; e.g., “I feel like crying many days”). The raw scores on the five subscales for negative mood, interpersonal difficulties, negative self-esteem, ineffectiveness, and anhedonia are transformed to age and gender-normed scores. The total CDI score ranges from 0 to 54 with higher scores indicating more symptoms of depression [29]. The CDI has good validity and acceptable reliability in children age 7 years and older [30]. Based upon published reliability and validity, the extent of use in the published literature, and expert panel consensus, an evidence-based review classified the CDI as “well-established” [31]. Due to its strong psychometric properties and widespread use in previously published pediatric pain studies, the PedIMMPACT has also recommended the CDI for assessing older child and adolescent depression [32]. The CDI has also been used in other pediatric chronic pain populations, facilitating research study group comparisons [33].

#### 2.4.4. Anxiety: Revised Children's Manifest Anxiety Scale

The Revised Children's Manifest Anxiety Scale, Second Edition (RCMAS-2) is a 49-item yes/no questionnaire that assesses symptoms of anxiety in children and adolescents from 6 to 19 years old [34]. Four subscales on the RCMAS-2 assess physiological anxiety worry, social anxiety, and defensiveness. The RCMAS-2 contains 40 items (yes/no) related to anxiety. The raw Total Anxiety score on the RCMAS-2 ranges from 0 to 40 with higher scores indicating more symptoms of anxiety [34]. The RCMAS-2 is an updated version of the original RCMAS. Based upon its published reliability and validity and the extent of use in the published literature, a Society of Pediatric Psychology (SPP) panel classified the RCMAS as “well-established” and the PedIMMPACT supported the use of the RCMAS [29, 31, 32, 35, 36]. The RCMAS has been applied in a study examining associations between social desirability and self-report of pain, disability, and psychological distress (depression, anxiety, and somatic symptoms) in a sample of children presenting to a multidisciplinary pediatric chronic pain clinic [37].

#### 2.4.5. Pain Coping: Pain Coping Questionnaire

The Pain Coping Questionnaire (PCQ) is a 39-item self-report tool especially designed for young people ages from 8 to 18 years old with pain [38, 39]. Using a 1 to 5 Likert scale, the PCQ assesses eight specific pain coping strategies: information seeking; problem solving; seeking social support; positive self-statements; behavioral distraction; cognitive distraction; externalizing; and internalizing/catastrophizing. Its authors have reported good validity and adequate reliability across the pediatric age range [38]. Higher-order factor analyses have indicated that the eight PCQ subscales load onto three higher-order factors (scales) [38]. The Approach Scale (comprised of the information seeking, problem solving, seeking social support, and positive self-statements subscales) measures direct attempts to deal with the pain and the use of active methods to regulate feelings when in pain. The Problem-Focused Avoidance Scale (comprised of the positive self-statements, behavioral distraction, and cognitive distraction subscales) measures attempts to disengage from the pain. The Emotion-Focused Avoidance Scale (comprised of the externalizing and internalizing/catastrophizing subscales) measures strategies in which emotions are freely expressed and strategies that reflect a lack of effort to regulate feelings when in pain [38]. Items on subscales and three higher-order scales are summed and then averaged to create PCQ score ranging from 1 to 5 [39]. An expert panel and evidence-based review under the aegis of the Society of Pediatric Psychology identified the Pain Coping Questionnaire (PCQ) as well-established [40]. The PCQ has been well-adopted [29, 41], and thus, other pediatric chronic pain sample data are available for comparison, particularly for juvenile rheumatoid arthritis and juvenile fibromyalgia [42–46].

### 2.5. Parent Measurement Instruments

#### 2.5.1. Parent Chronic Pain Intensity: Brief Pain Inventory-Short Form (BPI-SF)

The Brief Pain Inventory-Short Form (BPI-SF) is a self-administered questionnaire about the severity of adult pain and the impact of pain on daily function (i.e., functional disability) [47]. Though developed for cancer pain, the BPI-SF has demonstrated sufficient reliability and validity with chronic nonmalignant pain [48]. Respondents rate their pain intensity on BPI-SF using an 11-point numerical rating scale with anchors of “no pain” and “pain as bad as you can imagine” [49]. The BPI-SF also measures pain interference with seven daily activities, specifically, general activity, walking, work, mood, enjoyment of life, relations with others, and sleep [47]. Respondents rate each activity on an 11-pont scale (from 0, “does not interfere,” to 10, “completely interferes”). The BPI-SF pain interference value was scored here typically as the mean of the seven interference items, with a scoring range of 0 to 10 [50]. In this study, parents completed both the pain intensity and pain interference components of BPI-SF on themselves.

#### 2.5.2. Parent Depression: Beck Depression Inventory-Second Edition

In order to facilitate a similar parallel assessment of coexisting depression in the pediatric clinical patient and his/her parent, the Beck Depression Inventory-Second Edition (BDI-II) was applied. The BDI consists of 21 items, each scored on a 0 to 3 scale, with a total score range of 0 to 63. Higher total scores indicate more severe depressive symptoms; 0–13: minimal depression; 14–19: mild depression; 20–28: moderate depression; and 29–63: severe depression [51, 52]. The BDI-II has well-documented validity and reliability and has been widely utilized in research and clinical care [53, 54]. The Children's Depression Inventory (CDI) is a downward extension of the adult BDI [31].

#### 2.5.3. Parent Anxiety: Adult Manifest Anxiety Scale

In order to facilitate a similar parallel assessment of coexisting anxiety in the pediatric clinic patient and his/her parent, the Adult Manifest Anxiety Scale (AMAS) was applied. The AMAS is a 36-item (yes/no) self-report measure designed to assess anxiety in the adult population (18–59 years) [55, 56]. Three scales and 30 items on the AMAS assess worry/oversensitivity, social concerns/stress, and physiological anxiety. The raw Total Anxiety score on the AMAS ranges from 0 to 30, with higher scores indicating more symptoms of anxiety [56]. The AMAS has been shown to have excellent validity and reliability [55].

#### 2.5.4. Parent Stress: Parenting Stress Index/Short Form

The Parenting Stress Index/Short Form (PSI/SF) was derived from a full-length and rather burdensome parental stress index measure [57]. The abridged PSI/SF has been widely applied as a screening instrument for parents who are experiencing stressors [58]. The PSI/SF (3rd edition) consists of three subscales of 12 items each, with the 36 items rated on a five-point Likert scale. The subscales scores are totaled to create an overall rating of parents' stress levels related to parenting, ranging from 36 to 180 [29]. The PSI/SF has excellent validity for parenting stress across a range of chronic adversity conditions, including chronic health complaints and chronic pain disorders [58, 59].

### 2.6. Mapping of Study Variables into the Biobehavioral Model of Pediatric Pain

**Figure 2:**
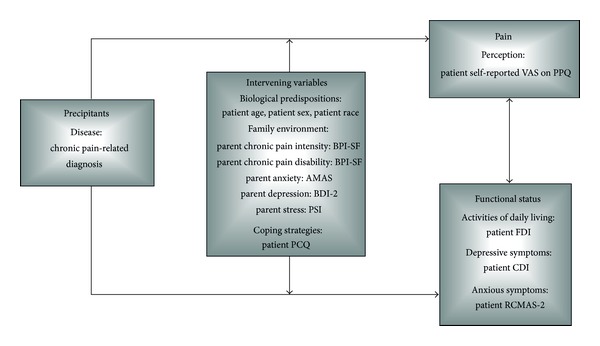
Study variables mapped into the modified Biobehavioral Model of Pediatric Pain [12].

The present study variables (Sociodemographics and Clinical History, Child/Adolescent Measurement Instruments, and Parent Measurement Instruments) were mapped into the Varni's Biobehavioral Model of Pediatric Pain (Figure 2), specifically into its four main components (precipitants, intervening variables, pain, and functional status).

### 2.7. Statistical Analyses

Continuous variables were summarized using descriptive statistics (mean and standard deviation or median and interquartile range). Categorical variables were summarized using frequency counts and percentages. In addition to generating Q-Q plots, the Shapiro-Wilk test was applied to confirm the normality of the present continuous variables and thus fitness for a parametric test statistic. Any continuous dependent outcome variable found to lack normality was analyzed with nonparametric statistics.

The relationships between patient self-reported functional disability (FDI), patient chronic pain intensity (PPQ), patient anxiety (RCMAS-2), patient depression (CDI), patient pain coping (PCQ Approach Scale, PCQ Problem-Focused Avoidance Scale, and PCQ Emotion-Focused Avoidance Scale), parent chronic pain intensity (BPI-SF Pain Intensity Subscale), parent functional disability (BPI-SF Pain Interference Subscale), parent anxiety (AMAS), parent depression (BDI-II), and parent stress (PSI) were assessed within the total sample using Spearman's correlation coefficients. Variables demonstrating a correlation coefficient of at least 0.30 with patient self-reported pain intensity (PPQ) and patient self-reported functional disability (FDI) were selected for inclusion in subsequent regression analyses.

Based upon their observed correlations, a multivariable linear regression model was then used to evaluate within the entire study sample the association between patient self-reported pain intensity (PPQ) and a subset of two of the above psychosocial variables: patient anxiety (RCMAS-2) and patient depression (CDI).

Based upon their observed correlations, a multivariable linear regression model was then used to evaluate within the entire study sample the association between patient self-reported functional disability (FDI) and a subset of seven of the above independent psychosocial variables: patient chronic pain intensity (PPQ), patient anxiety (RCMAS-2), patient depression (CDI), patient pain coping (PCQ Approach Scale and PCQ Emotion-Focused Avoidance Scale), parent chronic pain intensity (BPI-SF Pain Intensity Subscale), and parent functional disability (BPI-SF Pain Interference Subscale).

Specifically, given the exploratory nature of the analysis, a stepwise (forward entry) method, with entry criterion of *P* ≤ 0.05 and removal criterion of *P* ≥ 0.10, was applied. Based upon published evidence that sociodemographic factors are significantly associated with several pain-related characteristics in children with chronic pain, the two exploratory regression models also included patient age (years), patient gender (female or male), and patient race (African-American or Caucasian) [60].

Rather than relying on the default listwise deletion method for missing values, the mean of a variable for all other cases was used for imputing missing values in our dataset, the frequency of which ranged from 0% to 12%. Simple mean imputation was applied because it reportedly affects relationships between variables by conservatively “pulling” estimates of the correlation toward zero (bias toward the null value) [61]. However, a sensitivity analysis was performed with the cases of missing values deleted, and no differences were observed in the significant correlation and regression coefficients. *P* values <0.05 (two-sided) were considered significant. All statistical analyses were performed using IBM SPSS (Version 20.0).

## 3. Results

A total of 99 of the 145 eligible patients (68%) and their accompanying parents were enrolled in this study. The majority of the study participants in this cross-sectional, convenience sample were female (71%), early adolescents (mean age of 13.2 years, SD of 2.4). Eighty-one percent were Caucasian, 17% African-American, 1% Native American, and 1% Asian-American. The enrolled patients presented with a variety of primary chronic pain conditions: headache (21%); cervical, thoracic, lumbar, and/or sacral spine pain (19%); abdominal pain (18%); extremity or large joint pain (18%); fibromyalgia or a myofascial pain syndrome (15%); or peripheral neuropathic pain (including complex regional pain syndrome, CRPS, types I and II) (8%). All study patients had experienced their presenting pain condition for more than one month (median duration of 15 months, interquartile range of 7–36 months). Table 1 presents the mean (SD) values for the patient self-reported and parent self-reported scores on the applied study questionnaires, diagnostic instruments, and health surveys.

No data were collected on the reasons for study nonparticipation. However, the relatively low participation rate appeared to be due to frequently expressed parental time constraints and the already extensive evaluation performed on all new clinic patients. If both parents were available, one volunteered to serve as the sole study participant. Of the presently enrolled parental study participants and survey respondents, 93 (94%) were the patient's mother.

### 3.1. Relationship between Patient Self-Reported Pain Intensity, Patient Self-Reported Functional Disability, and Psychosocial Variables

Table 2 presents the Spearman's correlation coefficients between patient chronic pain intensity (PPQ), patient functional disability (FDI), patient anxiety (RCMAS-2), patient depression (CDI), patient pain coping (PCQ Approach Scale, PCQ Problem-Focused Avoidance Scale, and PCQ Emotion-Focused Avoidance Scale), parent chronic pain intensity (BPI-SF Pain Intensity Subscale), parent functional disability (BPI-SF Pain Interference Subscale), parent anxiety (AMAS), parent depression (BDI-II), and parent stress (PSI).

Significant correlation coefficients were observed between patient chronic pain intensity (PPQ) and patient anxiety (RCMAS-2), patient depression (CDI), patient pain coping (PCQ Approach Scale and PCQ Emotion-Focused Avoidance Scale), parent chronic pain intensity (BPI-SF Pain Intensity Subscale), and parent functional disability (BPI-SF Pain Interference Subscale).

Significant correlation coefficients were observed between patient functional disability (FDI) and patient chronic pain intensity (PPQ), patient anxiety (RCMAS-2), patient depression (CDI), patient pain coping (PCQ Approach Scale and PCQ Emotion-Focused Avoidance Scale), parent chronic pain intensity (BPI-SF Pain Intensity Subscale), parent functional disability (BPI-SF Pain Interference Subscale), parent anxiety (AMAS), parent depression (BDI-II), and parent stress (PSI).

### 3.2. Predictors of Patient Self-Reported Pain Intensity and Functional Disability

The results of the linear regression modeling indicated that patient self-reported pain intensity (PPQ) was significantly associated with patient anxiety (RCMAS-2) (Table 3) whereas patient self-reported functional disability (FDI) was significantly associated with patient chronic pain intensity (PPQ), patient anxiety (RCMAS-2), patient pain coping (PCQ Approach Subscale), and parent functional disability (BPI-SF Pain Interference Subscale) (Table 3).

## 4. Discussion

A biopsychosocial approach to the management of pediatric chronic pain has historically been widely emphasized [10, 62–69]. The biopsychosocial assessment and treatment of pediatric chronic pain emphasize not only the patient's personal experiential perspective but also the parent's own experiential perspective and in turn the interaction and dynamic within the parent-child dyad [2, 6, 70–75]. A substantial body of the literature has focused on the relationship between pediatric and parental anxiety, depression, somatization, coping, pain intensity, functional disability, and health-related quality of life [29, 33, 37, 44–46, 76–81]. However, the clinical utility of our present findings and much of these previously published data may be tenuous.

Psychological therapies have been widely studied for the management of chronic and recurrent pain in children and adolescents [82, 83]. However, in pragmatic terms, in an actual outpatient clinic setting, the ability to successfully address these cognitive, behavioral, and social issues can be challenging if neither the patient nor the parent are prepared to accept their presence and importance [84, 85]. Such reluctance often stems from the stigma attached to mental healthcare and the adverse reaction that the patient's chronic pain condition is somehow less legitimate (“real”) if it is not predominantly or exclusively physical (somatic) in origin and nature. Certainly, in the general community, access to a capable and engaging clinical psychologist or social worker, as well as insurance coverage for such services, while crucial, is often lacking. The end result is patient and parent disaffection, lack of adherence to treatment recommendations, and clinical loss to follow up [69, 86].

Moreover, despite seemingly effectively addressing and ameliorating these cognitive and behavioral issues, a pediatric patient's self-reported and the parent's proxy-reported levels of the child's pain intensity and functional disability can remain high. In such refractory and often enigmatic cases, terms like “family sickness model,” “parental enmeshment,” “pain amplification syndrome,” “Munchhausen's syndrome by proxy,” and “super girl syndrome” are used in clinical parlance but are ill-defined and difficult to measure.

Not surprisingly, while data are readily available on the prevalence and natural history of pediatric chronic pain [87–92], rigorous longitudinal studies on patient chronic pain management strategies are generally lacking [93–95]. This void leaves the often solo, lone pediatric pain medicine practitioner unsure as to what constitutes current, evidence-based best practices. Further research is needed to understand the complex biobehavioral processes involved in the development and the maintenance of chronic pain [69]. Development of novel, targeted biomedical and psychosocial therapies as well as comparative studies of existing treatments will help to improve treatment of pediatric chronic pain [69].

Pediatric pain is a highly individual and abstract concept, but it is also a tangible public health concern [96]. There appears to be a need for a more unified approach to pediatric pain management, which extends beyond the dedicated, yet few boutique pediatric pain medicine clinics or programs [93]. This more unified approach needs to take a more systems-level approach and include other pediatric community stakeholders like general pediatricians, school personnel, health policy makers, and elected officials [97–100]. It also needs to take into consideration the likely underrepresented and this undertreated minority and lower socioeconomic pediatric population, whose parents lack the wherewithal to access a dedicated pediatric pain medicine clinic or program [101].

### 4.1. Limitations

In the setting of chronic pain, parents and children may develop a shared narrative that is often repeatedly rehearsed in their successive medical encounters. This would include, in particular, ratings of pain intensity and functional disability. While our study patients and their parents were specifically instructed to complete all of the study questionnaires independently, they were not placed in separate rooms. Thus, there may have been further informant cross-contamination. Limitations of our study also include the relatively small sample size; however, it was comparable to that of other published pediatric chronic pain patient samples. A similar demographic and clinical profile has been observed during the last decade among patients referred to four other tertiary-care, multidisciplinary pediatric pain medicine clinics in the United States [29, 33, 102, 103]. In contrast, the present sample included a greater percentage of African-Americans, reflecting the demographics of the catchment area of the Children's Hospital of Alabama. Despite this greater percentage of African-American participants, minorities (especially ethnic Hispanics or Latinos) were underrepresented in this study, reducing the external validity of its findings. Lastly, our study sample was drawn from an anesthesiology-based, multidisciplinary pediatric chronic pain medicine clinic, and therefore, our present findings may have limited applicability (external validity) in other pediatric subspecialty or primary care practices and patient populations.

### 4.2. Conclusions

We observed significant correlations between pediatric *chronic pain intensity* and patient anxiety, patient depression, patient pain coping, parent chronic pain intensity, and parent functional disability. Pediatric chronic pain intensity was significantly associated with patient anxiety. We also observed significant correlations between pediatric *functional disability* and patient chronic pain intensity, patient anxiety, patient depression, patient pain coping, parent chronic pain intensity, parent functional disability, parent anxiety, parent depression, and parent stress. Pediatric functional disability was significantly associated with patient chronic pain intensity, patient anxiety, patient pain coping, and parent functional disability. These findings provide further empirical support to the multidimensional Biobehavioral Model of Pediatric Pain (Figure 1) that was developed by Varni and colleagues [12]. These findings are also consonant with the factors, identified in a Delphi poll of professionals with a specific interest in chronic pain in children and adolescents, as being associated with pediatric chronic pain and disability [7].

## Figures and Tables

**Table 1:** Measurement instruments completed by study patients and their parents.

Measurement instrument	Respondent	Value* (*N* = 99)
Pediatric Pain Questionnaire (PPQ)	Patient self-reported	51.8 (30.9)
Functional Disability Inventory (FDI)	Patient self-reported	23.0 (13.1)
Revised Children's Manifest Anxiety Scale (RCMAS-2)	Patient self-reported	15.6 (9.5)
Children's Depression Inventory Form (CDI)	Patient self-reported	12.5 (8.5)
Patient Pain Coping Questionnaire (PCQ)	Patient self-reported	
PCQ Approach Subscale		2.7 (0.7)
PCQ Problem-Focused Avoidance Scale (PFA) Subscale		2.7 (0.7)
PCQ Emotion-Focused Avoidance (EFA) Subscale		2.3 (0.8)
Brief Pain Inventory (BPI) Pain Intensity Subscale	Parent self-reported	3.2 (2.8)
Brief Pain Inventory (BPI) Pain Interference Subscale	Parent self-reported	3.2 (2.8)
Adult Manifest Anxiety Scale (AMAS)	Parent self-reported	12.9 (7.6)
Beck Depression Inventory-Second Edition (BDI-II)	Parent self-reported	11.8 (9.9)
Parenting Stress Index/Short Form (PSI)	Parent self-reported	50.6 (33.3)

*Mean (SD).

**Table 2:** Correlation coefficients between the study variables mapped into the Biobehavioral Model of Pediatric Pain [12].

Spearman *r* and *P* value	BDI-II Total Score	BPI Pain Intensity Score	BPI Pain Interference Score	CDI Total Score	PCQ Patient Approach Score	PCQ Patient PFA Score	PCQ Patient EFA Score	PSI Total Score	RCMAS-2 Total Score	*PPQ Patient Score *	*FDI Patient Score *
AMAS Total Score	**0.78** **<0.001**	**0.30** **0.002**	**0.32** **0.001**	0.17 0.10	0.079 0.44	−0.051 0.62	0.17 0.085	**0.51** **<0.001**	**0.23** **0.023**	0.052 0.61	**0.23** **0.025**
BDI-II Total Score	—	**0.43** **<0.001**	**0.40** **<0.001**	**0.33** **0.001**	0.014 0.90	−0.083 0.42	**0.27** **0.008**	**0.55** **<0.001**	**0.42** **<0.001**	0.09 0.38	**0.29** **0.004**
BPI Pain Intensity Score	—	—	**0.65** **<0.001**	**0.24** **0.018**	−0.012 0.90	−0.11 0.28	**0.29** **0.004**	**0.24** **0.019**	**0.35** **<0.001**	**0.26** **0.010**	**0.30** **0.003**
BPI Pain Interference Score	—	—	—	**0.28** **0.004**	0.003 0.98	−0.11 0.29	**0.28** **0.005**	**0.27** **0.007**	**0.34** **0.001**	**0.27** **0.007**	**0.37** **<0.001**
CDI Total Score	—	—	—	—	−0.039 0.70	**−0.25** **0.013**	**0.47** **<0.001**	**0.25** **0.012**	**0.64** **<0.001**	**0.30** **0.002**	**0.41** **<0.001**
PCQ Patient Approach Score	—	—	—	—	—	**0.50** **<0.001**	0.14 0.18	−0.026 0.80	0.043 0.67	**0.27** **0.008**	**0.31** **0.002**
PCQ Patient PFA Score	—	—	—	—	—	—	−0.19 0.056	−0.085 0.41	**−0.23** **0.023**	−0.039 0.70	−0.13 0.20
PCQ Patient EFA Score	—	—	—	—	—	—	—	0.19 0.060	**0.42** **<0.001**	**0.27** **0.006**	**0.35** **<0.001**
PSI Total Score	—	—	—	—	—	—	—	—	**0.29** **0.003**	0.071 0.48	**0.21** **0.033**
RCMAS-2 Total Score	—	—	—	—	—	—	—	—	—	**0.31** **0.002**	**0.39** **<0.001**
*PPQ Patient Score *	—	—	—	—	—	—	—	—	—	—	**0.45** **<0.001**

*Child/adolescent measurement instruments*:

Patient pain intensity: Pediatric Pain Questionnaire (PPQ).

Patient functional disability: Functional Disability Inventory (FDI).

Patient anxiety: Revised Children's Manifest Anxiety Scale-Second Edition (RCMAS-2).

Patient depression: Children's Depression Inventory Form (CDI).

Patient pain coping: Patient Pain Coping Questionnaire (PCQ)

PCQ Approach subscale.

PCQ Problem-Focused Avoidance Scale (PFA) subscale.

PCQ Emotion-Focused Avoidance (EFA) Subscale.

*Parent measurement instruments*:

Parent chronic pain intensity: Brief Pain Inventory (BPI) Pain Intensity Subscale.

Parent functional disability: Brief Pain Inventory (BPI) Pain Interference Subscale.

Parent anxiety: Adult Manifest Anxiety Scale (AMAS).

Parent depression: Beck Depression Inventory-Second Edition (BDI-II).

Parent stress: Parenting Stress Index/Short Form (PSI).

**Table 3:** Regression coefficients for significant predictors of patient self-reported pain intensity and self-reported functional disability. (Total sample (*N* = 99)).

Dependent variable	PPQ Score		FDI Functional Disability Score	
Independent variable	Beta-coefficient	*P* value	Beta-coefficient	*P* value
Patient chronic pain intensity (PPQ Pain Score)	—		0.104 (0.025, 0.18)	0.025
Patient anxiety (RCMAS-2 Score)	1.01 (0.37, 1.65)	0.002	0.31 (0.048, 0.57)	0.021
Patient pain coping (PCQ Approach Score)	—		4.22 (1.06, 7.38)	0.009
Parent functional disability (BPI-SF Pain Interference Score)	—		0.98 (0.12, 1.85)	0.027

FDI: Functional Disability Inventory; PPQ: Pediatric Pain Questionnaire; RCMAS-2: Revised Children's Manifest Anxiety Scale, Second Edition; PCQ: Pain Coping Questionnaire; BPI-SF: Brief Pain Inventory-Short Form.

Numbers in parentheses represent the 95% confidence intervals for reported point estimates.

## Abbreviations

PPQ: Pediatric Pain Questionnaire
VAS: Visual Analogue Scale
FDI: Functional Disability Inventory
CDI: Children's Depression Inventory Form
RCMAS-2: Revised Children's Manifest Anxiety Scale-2nd Edition
PCQ: Pain Coping Questionnaire
BPI-SF: Brief Pain Inventory-Short Form
AMAS: Adult Manifest Anxiety Scale
BDI-2: Beck Depression Inventory-2nd Edition
PSI: Parenting Stress Index.
